# Challenges in universal coverage and utilization of insecticide-treated bed nets in migrant plantation workers in Myanmar

**DOI:** 10.1186/1475-2875-13-211

**Published:** 2014-06-02

**Authors:** Myat H Nyunt, Khin M Aye, Myat P Kyaw, Thar T Kyaw, Thaung Hlaing, Kyaw Oo, Ni N Zaw, Thin T Aye, Nechi A San

**Affiliations:** 1 Department of Medical Research (Lower Myanmar), Yangon, Republic of the Union of Myanmar; 2 Department of Health, Naypyitaw, Republic of the Union of Myanmar; 3 Department of Medical Research (Upper Myanmar), Pyin Oo Lwin, Republic of the Union of Myanmar

**Keywords:** Malaria, ITN, LLIN, Migrant worker, Myanmar

## Abstract

**Background:**

High coverage of the bed nets can reduce mortality and morbidity of mosquito-borne diseases including malaria. Although the migrant workers are at high risk of malaria, there are many hidden challenges in universal coverage and utilization of the insecticide-treated nets (ITNs) in this populations.

**Methods:**

Cross sectional study was conducted in 170 migrant workers in palm oil plantation sites in Tanintharyi Region and 175 in rubber plantation sites in Mon State. A multistage stratified cluster sampling was applied to select the participants. During household visit, face-to-face interviews using structured pre-coded, pre tested questionnaires and direct observation on installation of the bed nets was conducted. Two focus group discussions in each site were done by sample stratified purposive sampling method mainly focused on effective utilization of bed nets.

**Results:**

Among them, 332 (96.2%) had a bed net and 284 (82.3%) had an ITN, while 204 (59.1%) had unused extranets. Among the ITNs users, 28.9% reported problems including insecticide smell (56.9%), dizziness (20.2%), headache (12.8%) and itchiness (9.2%). More than 75% received ITNs from health authorities and NGOs free-of-charge. More than 70% wanted to buy a net but they were unaffordable for 64% of them. On observation, only five families could show no bed net, but 80% showed 1–3 ITNs. Consistent utilization in all seasons was noted in 189 (53.1%), that was higher in palm oil plantation than rubber plantation workers (p = 0.0001) due to the nature of the work at night. Perceived malaria risk was also significantly higher ITNs consistent users than non-users (p = 0.0004) and better willingness to buy an ITN by themselves (p = 0.0005). They said that effectiveness of the ITNs was reduced after 6 months and 2–3 times washing. They wished to receive more durable smooth nets with small holes in lace. Misuses of the ITNs such as use the nets for animals and fishing, were also noted.

**Conclusion:**

There should be efforts to improve effective utilization of ITNs by continuous mass free distribution, durability monitoring, surveillance of insecticide resistance of the vector and behaviour change interventions in migrant plantation workers.

## Background

The goal established by the Member States at the World Health Assembly and the Roll Back Malaria (RBM) Partnership is to reduce malaria mortality and morbidity recorded in 2000 by 50% or more by the end of 2010 and by 75% or more by 2015 [1]. To reach this target, policy maker in endemic countries have to use effective strategies for mosquito control measures including supplying all persons at risk of malaria with insecticide-treated nets (ITNs) or indoor residual spraying [2,3]. High population coverage of the bed nets and effective treatment programmes may reduce the mortality and morbidity of malaria and other mosquito borne diseases up to 50% in the areas of intense perennial malaria transmission [3]. The World Health Organization (WHO) has recommended that universal coverage with long-lasting insecticidal nets (LLINs) should be achieved and maintained. In 2008, total 58 countries adopted the WHO recommendation to supply LLINs for all age groups at risk for malaria. This figure has represented an increase of 13 countries since 2007 [4].

One study suggested that free distribution of ITNs could save many more lives than cost-sharing programmes [5]. In 2006–2008, nearly 140 million LLINs were delivered to high burden countries in the African Region. However, it was noted that household ITN/LLINs ownership decreased by 13% and 37% after 24 and 36 months of mass distribution respectively indicating that routine distribution programme is important to maintain long-term coverage. WHO recommended routine monitoring of the durability of LLINs and the longevity of the insecticide to assess the requirements for ITNs maintenance [6].

There are many studies world-wide that found huge discrepancy between ownership versus use of ITNs. Studies quantified this difference as 95% *vs* 59% (Kenya) [7], 70% *vs* 53.1% (Nigeria) [8] and 90% *vs* 77% (Tanzania) [9] indicating household ownership was not reflected the utilization of the bed net.

**Figure 1:**
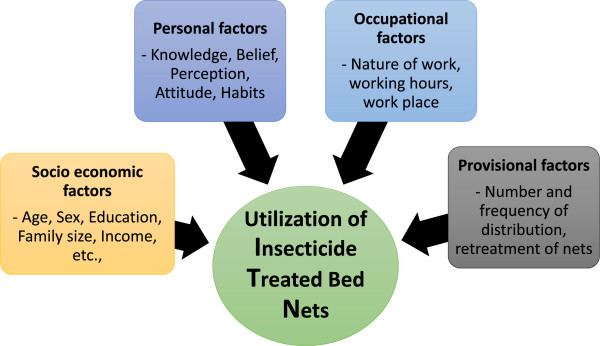
Conceptual frameworks for utilization of insecticide treated nets among migrant workers in plantation sites.

In Myanmar, number of ITNs distributed increased year by year starting from 2001 [4]. Malaria risk areas were prioritized for either free mass distribution of LLIN or treatment of the existing bed nets. In 2011, a total of 800,000 LLINs were distributed and 1,062,723 existing nets were treated. Similarly, the total numbers of 1,450,978 LLINs were distributed and 1,829,631 existing bed nets were treated in 2012 in malaria endemic areas [10]. These activities covered 5,627,445 populations in malaria high risk areas. The use of ITNs was thought to be appropriate for undeveloped and remote areas of the country where malaria control was difficult [11]. Challenges with ITNs still remain for replenishment of worn out nets, less efficacy and durability and abuse of nets especially in fishing areas [12]. Myanmar Artemisinin Resistance Containment (MARC) activities have been prepared and mass distribution of LLINs has been started since 2010, mainly focusing on migrant workers. Most of the migrant workers are non-immune and more vulnerable to malaria than local residents. Socioeconomic, personal, occupational and provisional factors influence the utilization of ITNs in migrant plantation workers [13] (Figure 1).

However, there is no documented study on coverage and utilization of ITNs in Myanmar migrant workers. To support the Myanmar Artemisinin Resistance Containment measures, the exploratory study on knowledge, distribution and utilization of ITNs in migrant plantation workers in Myanmar is cardinal.

## Methods

### Areas of study

This cross sectional descriptive study was carried out in Mon State and Tanintharyi Region in Myanmar. Mon State has a lot of rubber plantation sites where many migrant workers are employed and also has high morbidity of malaria. Similarly, many migrant workers from different parts of the country are employed in palm oil plantation sites in Kawthaung District, which has high malaria mortality and morbidity. In these sites, LLINs have been distributed by NGOs and the National Malaria Control Programme. Mass distribution of the LLINs has been distributed by the MARC Programme, in these study sites six months before conducting the survey [14].

### Participants in the study

Only adult migrant plantation workers were selected for the study. A migrant worker was defined as “a person who moves, but stays in one place for more than one month [15] with a view to being employed otherwise than on his own account”.

### Sample size

Utilization of the ITNs in all seasons was defined as sustainable use in this study. The sustainable use of ITNs among migrant workers had been found to be 10% in preliminary study in 2010. Therefore, assuming the sustainable use of ITNs among migrant population was 10%, 5% precision and 95% confident interval, non-response rate 5% (0.95), the required sample size was 146. To be representative, sample size was assumed to 160. It was multiplied by a design effect of two for multistage sampling [16]. Therefore, the sample size was 320. At least 160 respondents were selected from each of the study sites because each site has relatively the same population size in villages.

### Sampling procedure

A multistage stratified cluster sampling technique was used. Small villages with adult migrant population less than 60 were excluded to get comparable samples in both sites. Villages where migrant plantation workers were living, were screened and a total of 23 villages in Mon State and 20 villages in Tanintharyi Region were listed. From these, four villages were randomly selected. The list of adult migrant subjects was recorded in each of the villages. From these persons, systematic sampling procedures were applied. Sampling interval was calculated as total number of adult migrant subjects divided by 40. By adding sampling interval to the last respondents starting from the first persons, the study population was formulated. For example, if the migrant population was 80 in a village, sampling interval would be 2, and every 2^nd^ migrant workers in the list was selected. In this way, 40 respondents from each village i.e. 160 from each of the study sites were included in the study.

Data were collected by face-to-face interviews during household surveys (HHS) using pre-coded questionnaires and focus group discussions (FGD). The questionnaire was designed to collect information on population characteristics, education status, family size, net ownership, knowledge about mosquito nets, and source of nets, bed net utilization, total family members sleeping under nets, presence of unused nets in households and problems while using the net. There are three seasons, (summer, monsoon and winter) in Myanmar and its effect on bed net usage pattern was also assessed. Interviews were conducted by trained interviewers in the local languages using questionnaires adapted and modified from the WHO Malaria Indicator Survey (MIS) tools [17].

The questionnaire was pre-tested and finalized before the study. Moreover, observation of net installation was done in every respondent's household visit. Focus group discussions (FGD) were done at two places in each study site by stratified purposive sampling method. Group members were selected from rubber and palm oil plantation workers and their wives to obtain in depth information on effective utilization of bed nets in their population. Discussion guidelines were formulated as a series of open-ended questions.

### Data management

Data collected during the survey were checked and entered using Epi Info version 7. Analysis was performed using SPSS version 16. For univariate analysis, frequencies and proportions were calculated for household ownership and utilization of any net and ITNs, and cross-tabulated with background demographic characteristics of the households. Pearson's Chi squared test was used to determine association with a P-value of < 0.05 accepted as significant. Fisher's exact test was calculated for borderline significance and for cells with expected count less than five.

### Ethical consideration

All respondents were clearly informed on nature and purpose of the study, privacy question, benefits, and the right to refuse to participate or to withdrawal from the study and confidential handling of the data. Participation in this project was entirely voluntary. The interview was done non-working hours not to interference the participants' work. This project was obtained ethical clearance from the ethical committee of the Department of Medical Research (Lower Myanmar) (IERC_3/2010_21).

## Results

### Baseline demographic information

A total of 345 migrant plantation workers from both sites (170 in palm oil plantation sites in Tanintharyi Region and 175 in rubber plantation sites in Mon State) were included in this study. They were migrated from different parts of the country. People of working age (25–45 years) were the major respondents (54.78%, 189/345). Baseline demographic data were shown in Table 1.

**Table 1:** Demographic characteristics of the participants

**Category**	**Description**	** Mon State**		** Tanintharyi region**		** Both sites**	
		**Number**	**Percent**	**Number**	**Percent**	**Number**	**percent**
Age group	13-24 years	25	7.2	45	13.0	70	20.3
	25-34 years	57	16.5	57	16.5	114	33.0
	35-44 years	38	11.0	37	10.7	75	21.7
	>45 years	50	14.5	36	10.4	86	24.9
Sex	male	74	21.4	67	19.4	141	40.9
	female	96	27.8	108	31.3	204	59.1
Education status	No formal education	40	11.6	34	9.9	74	21.4
	Primary school level	79	22.9	91	26.4	170	49.3
	Middle and high school level	50	14.5	47	13.6	97	28.1
	Graduate	1	0.3	3	0.9	4	1.2
Family members	1 to 3	77	22.3	82	23.8	159	46.1
	4 to 6	78	22.6	75	21.7	153	44.3
	>6	35	10.1	33	9.6	68	19.7
Family income per month (USD)	<50	48	13.9	9	2.6	57	16.5
	50-79	49	14.2	28	8.1	77	22.3
	80-99	27	7.8	32	9.3	59	17.1
	100-150	41	11.9	43	12.5	84	24.3
	>150	5	1.4	63	18.3	68	19.7
Perceived risk on malaria	Yes	135	39.1	128	37.1	263	76.2
	No	35	10.1	47	13.6	82	23.8

### Bed net ownership and utilization pattern

Although 13 (3.8%) respondents had no bed net at all, only 15% (52/345) had no ITNs and 27% (94/345) had no untreated bed net in their household. Frequent washing (more than 12 times per year) was noted in 12.6%. More than half reported going to bed early 9 pm. The different seasons may influence the bed net utilization pattern, it was assessed in this study. More than 80% used bed nets in all seasons regularly but some never use the nets in summer. The main reason for utilization of ITNs was to prevent mosquito bites. Ownership and utilization of the bed nets are shown in Table 2.

**Table 2:** **Ownership *versus *** **utilization of the bed nets**

**Category**	**Description**	**Number**	**% of total**
ITN/LLIN ownership	0 net	52	15.1
	1 to 3 nets	273	79.1
	4 to 6 nets	20	5.8
Non ITN (untreated bed net) ownership	0 net	94	27.2
	1 to 3 nets	243	70.4
	4 to 6 nets	7	2.0
	> 6 nets	1	0.3
Washing of ITN/LLIN	no history of washing	146	42.3
	1 to 3 per year	61	17.7
	4 to 6 per year	45	13.0
	7 to 12 per year	49	14.2
	>12 per year	44	12.7
Time to go bed	Before 9 hour	202	58.5
	9 to 11 hour	129	37.4
	After 11 hour	5	1.4
	Irregular	9	2.6
Utilization pattern of different seasons	Monsoon only	3	0.9
	Monsoon and winter seasons only	12	3.4
	winter season only	2	0.6
	All seasons	281	81.4
	When the weather is cold	11	3.2
	When the mosquitoes are common	13	3.8
	Never	13	3.8
	Others	10	2.9
Reason for utilization	To prevent weather	22	6.38
	To prevent mosquito bites	313	90.7
	Others	77	22.3

### Reasons for non-utilization of ITNs

**Figure 2:**
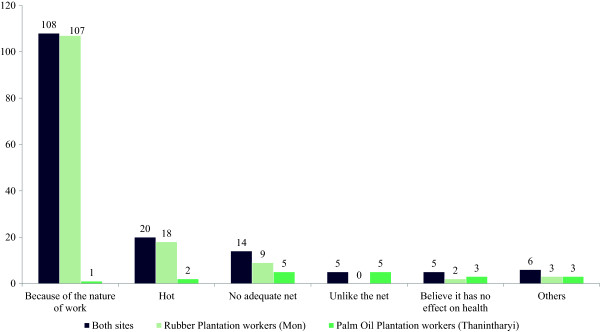
Reasons for not utilization of the ITN in migrant workers in 2 sentinel sites.

A total of 31.8% (110/345) said that they had to work at night and this factor was a major reason for non-utilization of ITNs during the whole night. Number of working hours varied among migrant workers (5.8 ± 2.4, mean ± SD) in rubber plantation sites at Mon State. Reasons for non-utilization of the ITNs were shown in Figure 2.

More than half, 59.1% (204/345) reported that they had unused nets and 33% had 1–2 unused extranets. Among the ITNs users, 100 (28.9%) reported problems while using ITNs, such as insecticide smell 62 (56.9%), dizziness 22 (20.2%), headache 14 (12.8%), itchiness 10 (9.2%) and difficulty in breathing 1 (0.9%).

Upon direct observation of the nets installed inside their homes, only five families could not show a bed net (ITN/LLIN or untreated net), but 80% showed one to three bed nets in the household. Distribution of the ITNs and their willingness to buy an ITN was shown in Table 3.

**Table 3:** Source and distribution of ITN and willingness to buy an ITN

**Category**	**Description**	**Number**	**Percent**
Source of ITNs	Buy from the market	3	0.9
	Local health authorities	203	58.8
	NGOs	65	18.8
	Other	22	6.4
Charges for ITNs	Received free of charge	288	83.5
	Bought	5	1.4
Affordable to buy ITN	Yes	124	35.9
	No	221	64.1
Like to buy ITN	Yes	256	74.2
	No	89	25.8
Number receiving ITN during free distribution	Not received	51	14.8
	1 time	224	64.9
	2 times	45	13.0
	More than 3 times	25	7.2
No. of ITN received	No nets	51	14.8
	1 net	121	35.0
	2 nets	103	29.9
	More than 3 nets	70	20.3
Methods or materials for retreatment	Yes	31	8.7
	No	313	87.9
Number of bed net observed in household	No net	5	1.5
	1 to 3 nets	274	80.1
	3 to 6 nets	62	18.1
	> 6 nets	1	0.3
History of fever within 2 weeks	Yes	39	11.3
	No	306	88.7

### Source of information on ITNs

Regarding the source of information on ITNs, health personnel were the major informers to the migrant workers (45.8%, 174/380) followed by community leaders (17.6%, 67/380) and, friend and family (16.8%, 39/380). It was noted that mass media including television, radio and newspapers have little role in information channels among these migrant workers.

### Consistent utilization of ITNs

Although ITN utilization was noted in most of the households, consistent utilization was noted in 53.1% (189/145). Consistent utilization of the ITNs was higher in palm oil plantation than rubber plantation workers (χ^2^ = 94.25, p = 0.0001). This was mainly due to the nature of the work at night among rubber plantation workers. Perceived malaria risk was also significantly higher in consistent ITN users than non-users (χ^2^ = 12.52, p = 0.0004). Consistent ITN users showed better willingness to buy an ITN than non-users (χ^2^ = 12.24, p = 0.0005). But consistent ITN users were less affordable to buy an ITN than non-user (χ^2^ = 26.37, p = 0.0001).

### Qualitative findings on utilization of ITNs

One of the 21-year old migrant women commented on distributed ITNs as

“I wish to receive the retreatment materials so that we can use ITNs consistently. It should be mentioned how to treat or soak the bed net with insecticide”.

A 50-year old migrant woman said

“*I don*’*t know that is ITN. I don't like it because it is too rough in texture with big pits. It looks like the nets used for animals such as buffalos and cows in my native town. Some of villagers use it to catch up fish”.*

36-year old migrant women also said

“I prefer CYC (Cotton 2-ply) bed net because these ITNs are not wind proof and cause dizziness, insecticide smell, and difficulty in breathing. So, ITNs were not currently used”.

Another 30-year old migrant rubber plantation worker mentioned how to prevent the mosquito bites as

“I used to collect the milk from rubber plants the whole night. The ITN distributed is almost useless for me. So, I use mosquito coils on the ladder, long clothes as protective wearing and apply repellent to prevent mosquito bites”.

One of the 35-year old respondent said

“The distributed ITN is not long lasting, because it was easily damaged after a few months. Its effectiveness is reduced after 2–3 time washing and now mosquitoes can rest on the net like non-ITN”.

A 50-year old migrant worker also expressed his experience as follows:

“Some men sleep outside of the bed net because they are not familiar with the bed net as it causes difficulty in breathing. So they fear to sleep inside a bed net and feel hot. Sometimes, a few men drink heavily and cannot control themselves and sleep outside the net”.

## Discussion

Indoor residual spraying (IRS) and insecticide-treated bed net (ITN) remain the frontline interventions for malaria vector control [18]. However, there are many factors contributing to the universal coverage and effective utilization of ITNs. A meta-analysis of household surveys done in Nigeria on net utilization and ownership found a wide gap between bed net ownership and utilization [19]. Moreover, utilization varied with seasons of the year. While 99% of the net recipients were found to use the nets during rainy season, only 20% was noted during the dry season [20]. Socio-demographic characteristics like age, education, size of household, and ethnicity were also found to influence the use of bed nets [21].

Free and mass distributions of the ITNs were done in both sentinel sites as one of the activities of the MARC Programme. However, sometimes migrant workers cannot use the ITN for the whole night because of the nature of their work. This was obviously noted in rubber plantation sites that need to do their work at night. Most of the ITN users used nets all seasons but some of them slept outside of the bed net because of the hot weather and their bad habits.

Most of migrant workers had heard of ITN only after free distribution. Therefore, the source of information on ITNs was mainly from health personnel, friends and family, and community leaders. Their low education level and lack of adequate facilities lead to difficulty in health education through mass media such as video, FM radio channel, TV programme and press media.

Free distribution was found as one of the major factors causing utilization of ITN in migrant workers. It found that 15% had no ITN and 27% had no untreated bed net. But consistent utilization was noted in just 53% of the respondents because of the nature of work and other behaviours.

**Figure 3:**
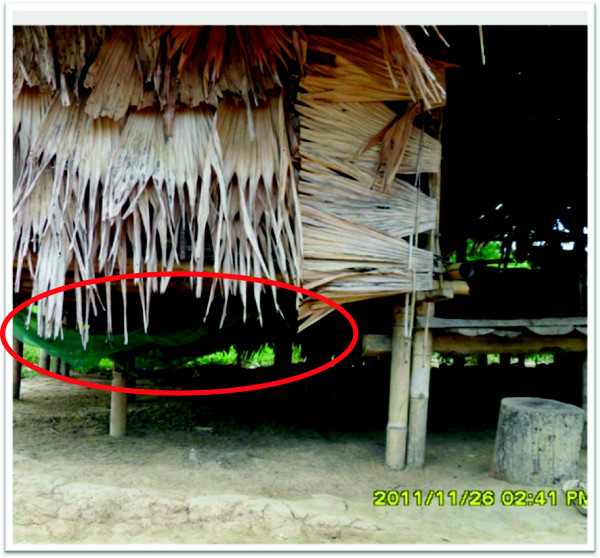
Misuse of the bed net for animal was observed in one of household visit.

Challenges with ITNs still remaining include replenishment of worn out nets, unknown efficacy and durability, and abuse of nets e.g. in fishing areas [12,22]. Misuses of the ITN, such as use to catch fish and use of nets for animals were also noted (Figure 3). Some of them also complain that after a few months of using the ITN, mosquito could adhere the net. This may be due to many factors [23,24] including insecticide resistant mosquitoes, poor quality of the nets, improper installation, frequent washing and drying in direct sunlight. It is important to exclude the insecticide resistant mosquitoes and find out the major factors causing the low effectiveness of the ITNs.

## Conclusion

Universal coverage remains the goal for all people at risk of malaria especially in migrant plantation workers. Free distribution of the ITN/LLINs is crucial to the success of maintaining universal coverage of bed nets in Myanmar. To further improve coverage and effective utilization of ITN/LLINs among migrant workers, regularly updated, interactive, comprehensive national advocacy, IEC/BCC activities, and operations research feeding into and providing timely and sound evidence to guide implementation and inform policy decision-making are critical. There should be efforts to improve the effectiveness of ITN and/or behaviour change interventions to improve net longevity and usage with continuous mass free distribution, durability monitoring and insecticide resistance of the vector in target migrant plantation workers.

### Policy implications and recommendations

•Net manufacturers and purchasers should be aware that the end users would like to receive more durable and reliable ITNs.

•Behaviour change communication and health promotion on effective utilization of the ITNs in target migrant population should be stressed.

•Integrated Vector Management should be emphasized, especially on the migrant employees who need to work at night.

•It is also recommended to monitor the efficacy and durability of the distributed ITNs and determine the factors responsible for reducing the efficacy of ITNs.

## Abbreviations

ITNs: Insecticide treated bed nets; LLINs: Long lasting insecticidal nets; BCC: Behaviour change communication; MARC: Myanmar Artemisinin Resistance Containment; HHS: Household survey; FGD: Focus group discussion; NGO: Non-Governmental Organization; USD: United State Dollar.

## Competing interests

The authors declare that they have no competing interests.

## Authors’ contributions

MHN, KMA, MPK, TH and TTK conceived and designed the study. MHN, KMA, NNZ, TTA and NAS conducted the field work. KMA, KO and MHN conducted statistical analysis, data validation and management. All authors contributed during writing, and read and approved the manuscript.
